# Supplementary material for: A megatransposon drives the adaptation of Thermoanaerobacter kivui to carbon monoxide
Source: Nat Commun. 2025 May 6;16:4217. doi: 10.1038/s41467-025-59103-8 (PMC12056078; doi:10.1038/s41467-025-59103-8)
Supplement: Supplementary file 1 — Supplementary Information [file 41467_2025_59103_MOESM1_ESM.pdf]

A megatransposon drives the adaptation of *Thermoanaerobacter kivui* to carbon monoxide

Hocq *et al.*

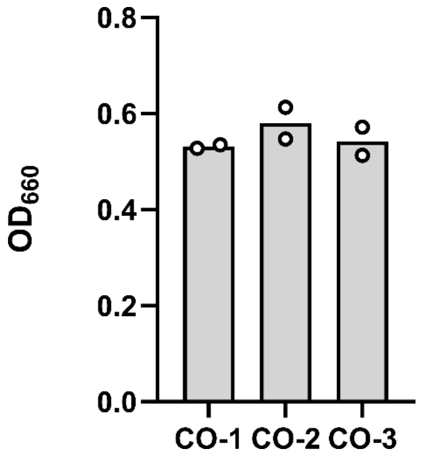

Supplementary Figure 1. Growth of the CO-1, CO-2 and CO-3 strains in serum bottle under 2 bar 100% CO. Serum bottles were inoculated at initial OD_{660} = 0.01. Maximal OD_{660} value is shown (CO-1: 5 days, CO-2: 7 days, CO-3: 14 days, n=2 independent experiments). Source data are provided as a Source Data file.

a.

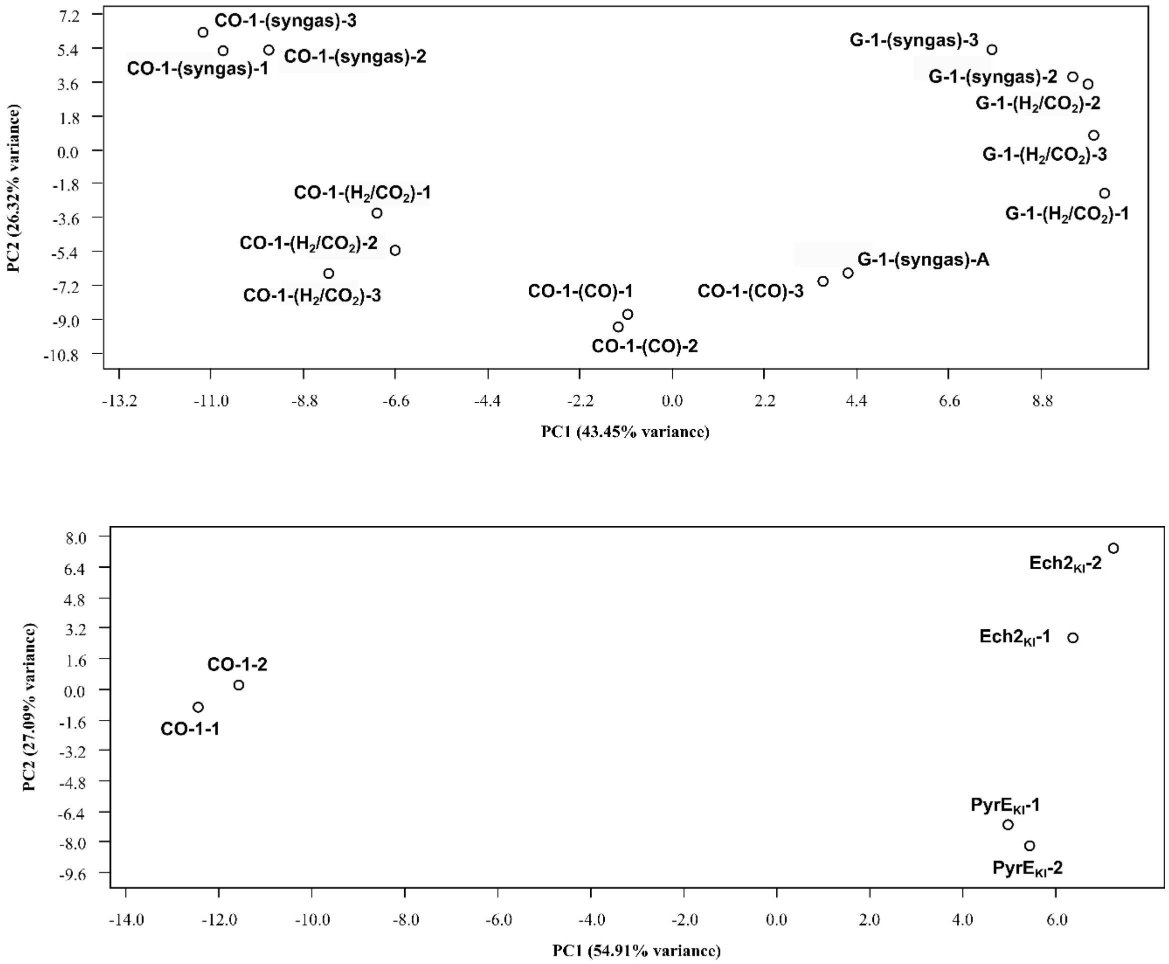

Supplementary Figure 2. Principal component analyses (PCA) of transcriptomic data. a. PC1/2 plot from steady-state transcriptomics data for the G-1 and CO-1 comparative experiment on H_2/CO_2, syngas and CO in bioreactors. b. PC1/2 plot for transcriptomics data for the PyrEKI, CO-1 and Ech2KI comparative experiment on syngas in serum bottles. PCA was generated with DESeq2 under default parameters.

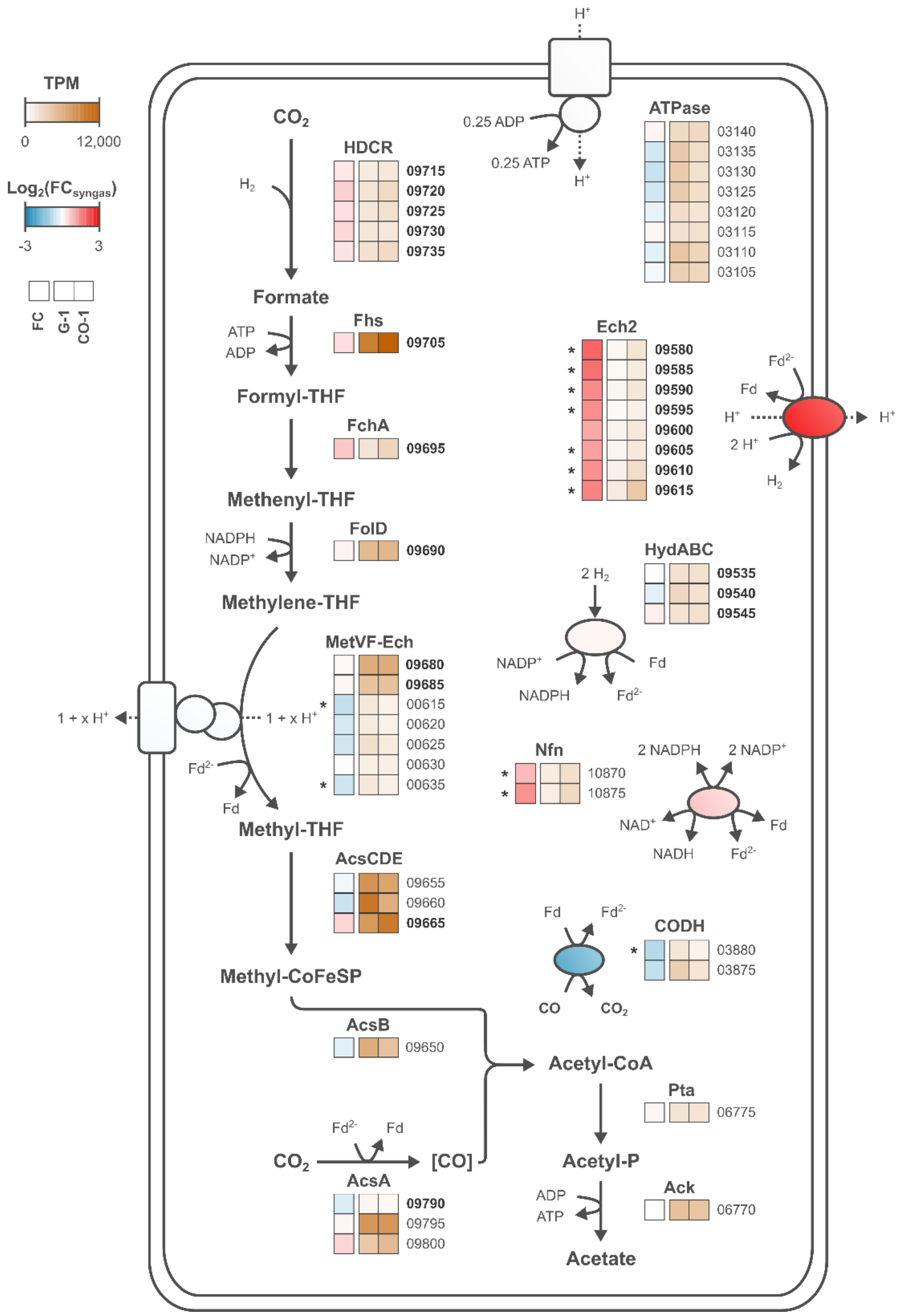

Supplementary Figure 3. Steady-state transcriptomics view of metabolism in G-1 and CO-1 strain. Central metabolism comprising the WLP as well as genes involved in energy conservation are shown. Mean TPM values as well as fold changes from differential expression analysis (DESeq2) for G-1 (syngas) and CO-1 (syngas) are described as a heat map. Locus tags in bold indicate genes present on Tn$_{CO-1}$. Asterisks correspond to statistically significant values (two-sided Wald test, Benjamini and Hochberg adjusted *p*-value < 0.05). RNA-seq was performed using RNA extracted from triplicate continuous fermentation experiments. Source data are provided as a Source Data file.

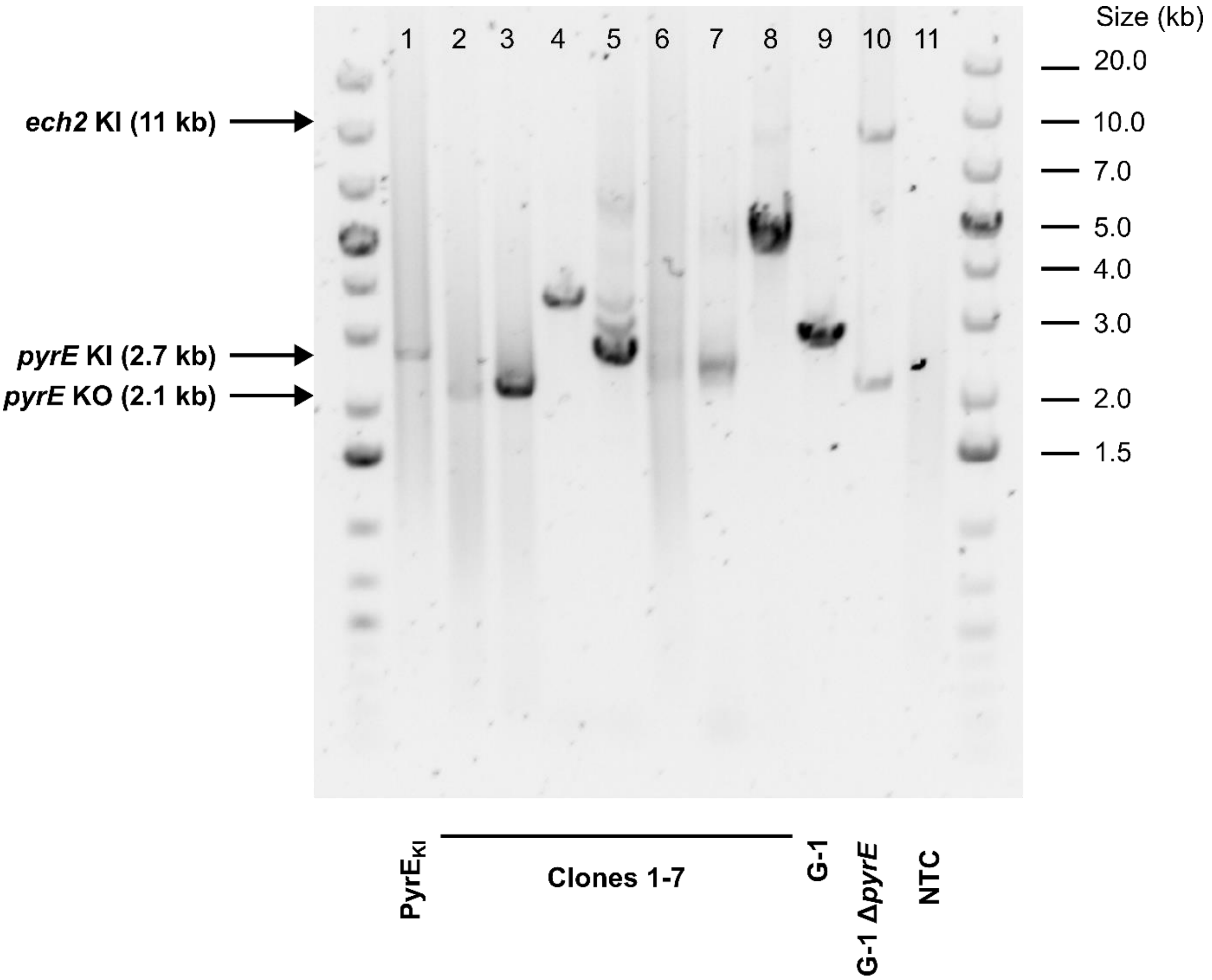

Supplementary Figure 4. Genotyping of clones obtained directly after transformation of the Ech2KI DNA template. PCR on gDNA targeting the *pyrE* locus. Controls: PyrEKI (KI of *pyrE* without *ech2* subunit genes), G-1 (WT), G-1 Δ*pyrE* (recipient strain), NTC: no template control. Expected sizes are indicated with an arrow. The genotyping of clones was performed as a single experiment. Source data are provided as a Source Data file.

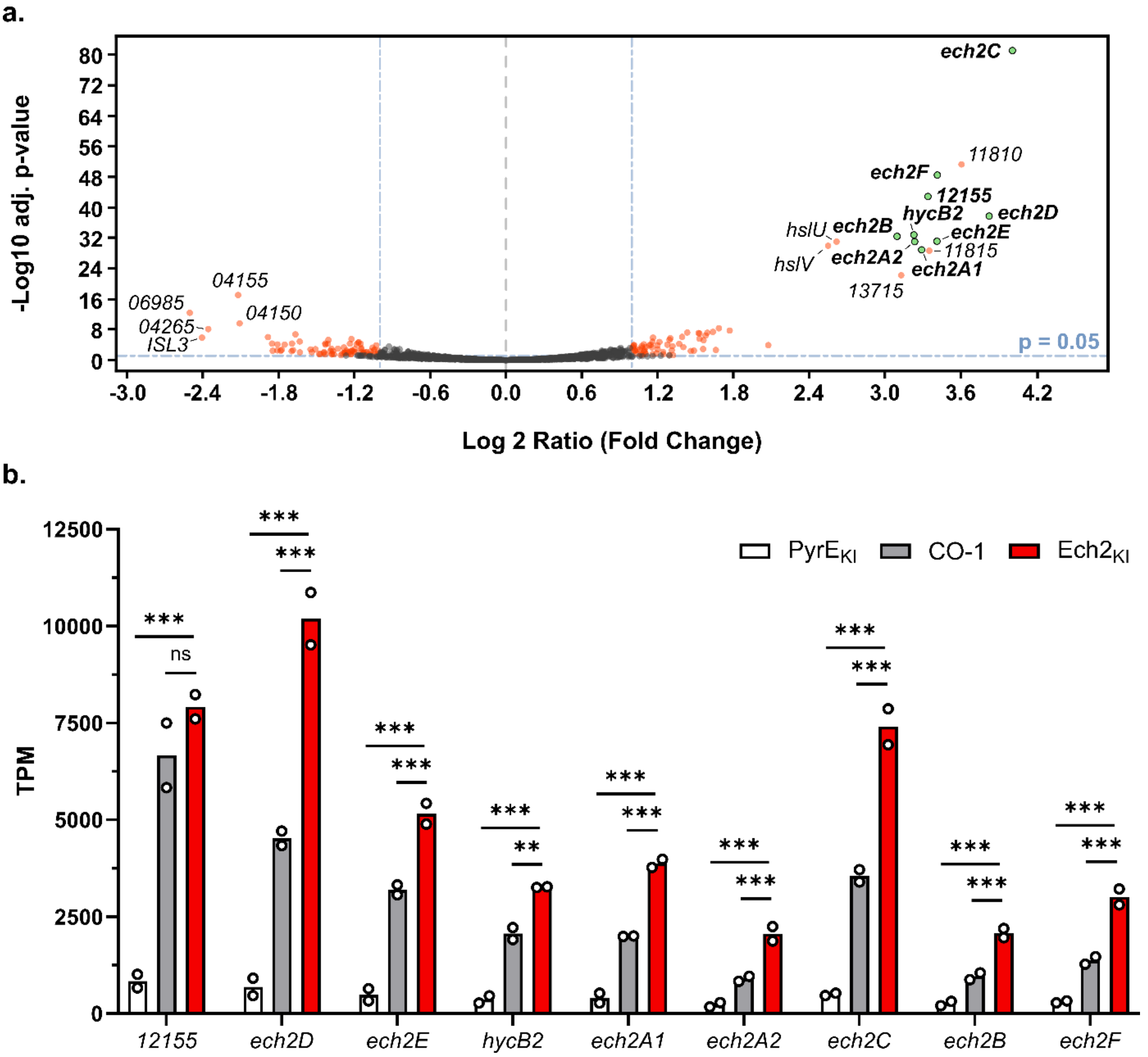

Supplementary Figure 5. *ech2* genes are the major upregulated genes in Ech2$_{KI}$. a. Volcano plot showing differentially expressed genes (two-sided Wald test, Benjamini and Hochberg adjusted *p*-value < 0.05, |Log$_2$(FC)| > 1, orange) in differential expression analysis (Ech2$_{KI}$ versus PyrE$_{KI}$ comparison is shown). *ech2* locus genes are highlighted in green. Raw data are shown in Supplementary Table 7. b. Transcriptomic snapshot of the *ech2* locus in the PyrE$_{KI}$, CO-1 and Ech2$_{KI}$ strains. TPM values as well as significance from differential expression analysis are shown. **: Benjamini and Hochberg adjusted *p*-value < 0.01; ***: adjusted *p*-value < 0.001; ns: not significant, adjusted *p*-value > 0.05. RNA was extracted from serum bottles experiments (n=2 independent experiments, log phase, under 3 bar CO/H_2/CO_2/N_2: 25/60/15/0). Source data are provided as a Source Data file.

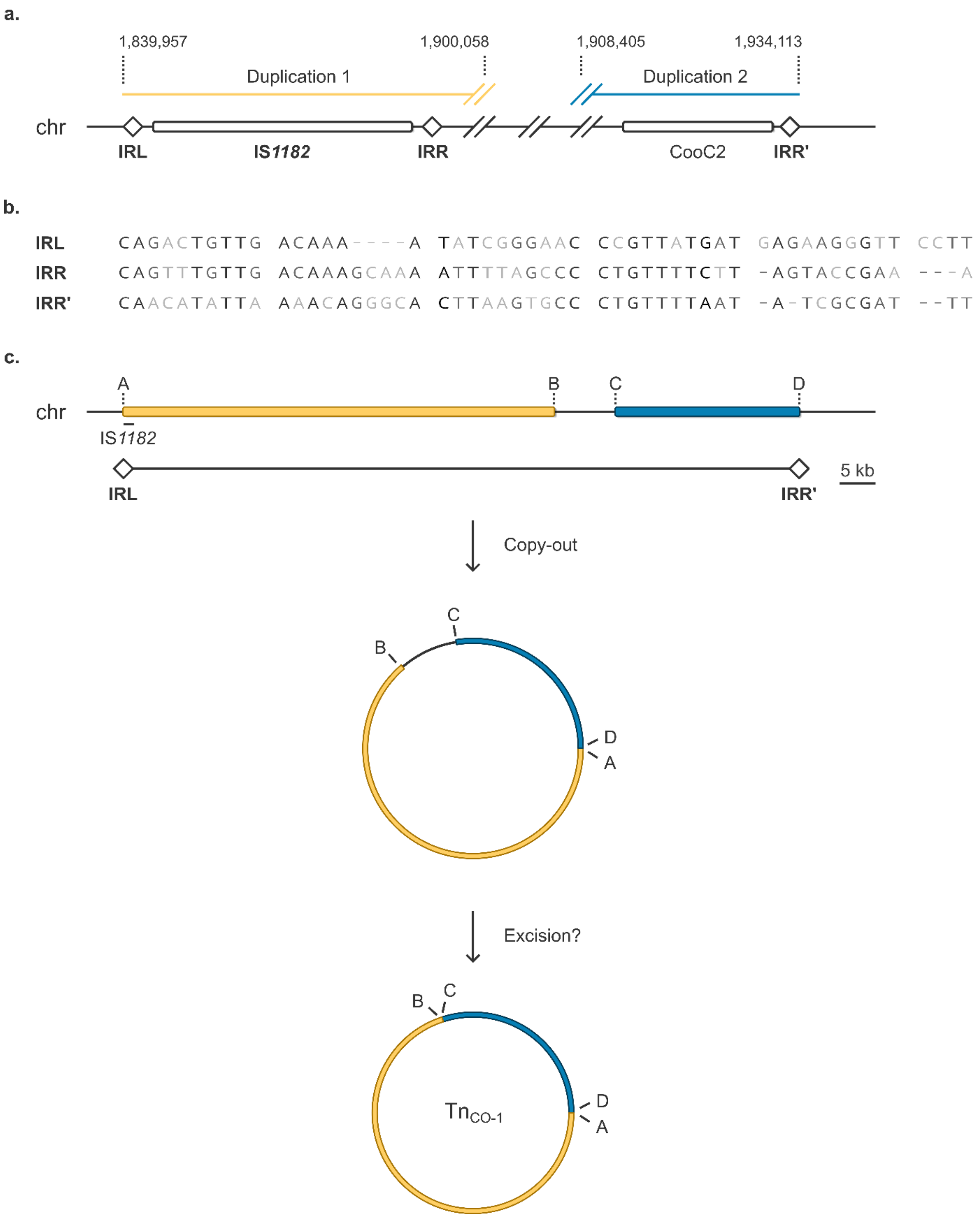

Supplementary Figure 6. Hypothetical mobilization process of Tn$_{CO-1}$. a. Putative IS*1182* inverted repeat positions on *T. kivui* chromosomal Tn$_{CO-1}$ origin region. IRL: inverted repeat left. IRR: inverted repeat right. IRR': alternative IRR. IRL and IRR were determined by homology to the *Caldanaerobacter subterraneus* IS*1182*-like IRL and IRR sequences on ISfinder (https://isfinder.biotoul.fr/). b. Alignment of IRL, IRR and IRR' sequences. c. Tn$_{CO-1}$ is proposed to originate from a copy-out mechanism involving IS$_{1182}$ and IRL/IRR' (represented at position A and D). Following or during this step, DNA comprised between positions B and C is excised by an unknown mechanism.

Supplementary Table 1. **Structural variations found in CO-1.** Positions refer to wild-type *T. kivui* (GCA_963971585.1).

Start	End	Type	Length (bp)	Comment
233,431	235,869	deletion	2,438	-
340,333	340,333	insertion	1,507	ISL3 transposon insertion, disrupts promoter of TKV_RS01665 (energy-coupling factor ABC transporter permease)
1,632,753	1,632,753	insertion	1,602	ISLre2 transposon insertion, disrupts promoter of TKV_RS08290 (sugar ABC transpoter permease)
2,101,807	2,103,409	deletion	1,602	ISLre2 transposon deletion

Supplementary Table 2. SNV / indel / SV analysis of the Ech2KI genome. Positions refer to wild-type *T. kivui* (GCA_963971585.1).

SNVs / indels				
**Position**	**Type**	**Locus tag**	**Effect**	**Annotation**
81,955	Substitution	TKV_RS00385	V111I (GTC → ATC)	fumarate hydratase subunit alpha FumA
139,602	Substitution	TKV_RS00690	G297R (GGA → AGA)	hydrogenase formation protein HypD
195,626	Substitution	Intergenic	Intergenic (G → A)	
331,571	Substitution	TKV_RS01615	A123V (GCG → GTG)	precorrin-3B C(17)-methyltransferase CobJ
341,995	Substitution	TKV_RS01675	Q207* (GAG → TAG)	cobalt ECF transporter T component CbiQ
535,193	Substitution	TKV_RS07920	D86A (GAC → GCC)	RNA polymerase sigma factor RpoD
535,714	Substitution	TKV_RS07920	E260K (GAG → AAG)	RNA polymerase sigma factor RpoD
625,455	Substitution	TKV_RS07415	A109P (GCT → CCT)	phosphate signaling complex protein PhoU
741,657	Insertion	TKV_RS06865	E111Efs*12 (GAG → GAGG)	Stp1/IreP family PP2C-type Ser/Thr phosphatase
774,815	Substitution	TKV_RS06680	G252E (GGG → GAG)	signal recognition particle protein
788,983	Substitution	TKV_RS06600	A167V (GCA → GTA)	GTP-sensing pleiotropic transcriptional regulator CodY
899,937	Insertion	TKV_RS05995	Y260Ifs*1 (TAT → ATAT)	stage V sporulation protein AD SpoVAD
1,140,288	Substitution	TKV_RS04760	R44K (AGG → AAG)	heat-inducible transcriptional repressor HrcA
1,548,987	Substitution	TKV_RS02645	V193L (GTC → CTC)	redox-sensing transcriptional repressor Rex
1,570,790	Substitution	TKV_RS02535	None (TCG → TCC)	ABC transporter permease
1,590,344	Deletion	TKV_RS08085	F267Ffs*4 (TTTT → TTT)	radical SAM protein
1,804,931	Substitution	TKV_RS09080	None (CCC → CCT)	gephyrin-like molybdotransferase Glp
1,963,781	Substitution	TKV_RS10045	D187E (GAT → GAA)	elongation factor Tu
1,968,320	Substitution	TKV_RS10070	I1012R (ATA → AGA)	DNA-directed RNA polymerase subunit beta' RpoC
2,030,568	Substitution	TKV_RS10360	None (CCG → CCT)	endospore germination permease
2,057,783	Substitution	TKV_RS10485	V342L (GTA → CTA)	DUF4914 family protein
2,134,406	Deletion	TKV_RS10875	P404Rfs*60 (CCACTGAAGGCT → CGT)	NAD+-dependent NADPH:Fd oxidoreductase, NfnB subunit
**Structural variations**				
**Start**	**End**	**Type**	**Length (bp)**	**Comment**
717,189	717,189	insertion	8,204	Ech2 KI
800,300	800,300	insertion	1,602	ISLre2 transposon insertion, disrupts TKV_RS06525 (disrupts flagellar basal body-associated FliL family protein)
1,372,036	1,372,036	insertion	1,602	ISLre2 transposon insertion, disrupts promoter of TKV_RS03520 (DUF2225 domain-containing protein)
1,632,753	1,632,753	insertion	1,602	ISLre2 transposon insertion, disrupts promoter of TKV_RS08290 (sugar ABC transpoter permease)
1,817,131	1,817,131	insertion	1,602	ISLre2 transposon insertion, intergenic
2,101,807	2,103,409	deletion	1,602	ISLre2 transposon deletion
2,129,549	2,130,905	deletion	1,356	IS30 transposon deletion
