# Supplementary material for: A megatransposon drives the adaptation of Thermoanaerobacter kivui to carbon monoxide
Source: Nat Commun. 2025 May 6;16:4217. doi: 10.1038/s41467-025-59103-8 (PMC12056078; doi:10.1038/s41467-025-59103-8)
Supplement: Supplementary file 3 — Description of Additional Supplementary Files [file 41467_2025_59103_MOESM3_ESM.pdf]

Description of Additional Supplementary Files

File Name: Supplementary Data 1
Description: SNV/indel analysis of the CO-1, CO-2 and CO-3 genomes. Positions refer to wild type *T. kivui* (GCA_963971585.1).

File Name: Supplementary Data 2
Description: Steady-state transcriptomics data and analysis of G-1 and CO-1 under H2/CO2 (G-1, CO-1), syngas (G-1, CO-1) and CO (CO-1) in bioreactors.

File Name: Supplementary Data 3
Description: Log-phase transcriptomics data and analysis of PyrEKI, Ech2KI and CO-1 under syngas in serum bottles. For differential expression analysis, log2(Fold Change) and adjusted *p*-values (two-sided Wald test, Benjamini and Hochberg correction) are shown.

File Name: Supplementary Data 4
Description: Strains, plasmids and primers used in this study. Carboxydotrophy is defined here as the ability to grow on CO as sole carbon/energy

File Name: Supplementary Data 5
Description: Overview of the sequencing files generated and used within the study.
